# Effects of a Gait Training Program on Spinal Cord Injury Patients: A Single-Group Prospective Cohort Study

**DOI:** 10.3390/jcm12237208

**Published:** 2023-11-21

**Authors:** Alexander Echemendía del Valle, Juan Enrique Bender del Busto, Armando Sentmanat Belisón, Juan Nicolás Cuenca-Zaldívar, Oliver Martínez-Pozas, Pedro Martínez-Lozano, Samuel Fernández-Carnero, Norberto Valcárcel Izquierdo, Eleuterio A. Sánchez-Romero

**Affiliations:** 1 Department of Therapeutic and Prophylactic Physical Education, Faculty of Physical Education, University of Physical Education and Sports Sciences, Havana 10600, Cuba; alexander.echemendia@infomed.sld.cu (A.E.d.V.);; 2 International Center for Neurological Restoration, Havana 11300, Cuba; jebender@infomed.sld.cu; 3 Grupo de Investigación en Fisioterapia y Dolor, Departamento de Fisioterapia, Facultad de Enfermería y Fisioterapia, Universidad de Alcalá, 28801 Alcalá de Henares, Spain; samuelfernandezcarnero@gmail.com; 4 Research Group in Nursing and Health Care, Puerta de Hierro Health Research Institute-Segovia de Arana (IDIPHISA), 28222 Majadahonda, Spain; 5 Physical Therapy Unit, Primary Health Care Center “El Abajón”, 28231 Las Rozas de Madrid, Spain; 6 Interdisciplinary Research Group on Musculoskeletal Disorders, Faculty of Sport Sciences, Universidad Europea de Madrid, 28670 Villaviciosa de Odón, Spain; oliver.martp@gmail.com; 7 Physiotherapy and Orofacial Pain Working Group, Sociedad Española de Disfunción Craneomandibular y Dolor Orofacial (SEDCYDO), 28009 Madrid, Spain; 8 Occupational Therapy, Rehabilitation and Physical Medicine, Escuela Internacional de Doctorado, Universidad Rey Juan Carlos, 28933 Alcorcón, Spain; 9 Department of Physiotherapy, Faculty of Sport Sciences, Universidad Europea de Madrid, 28670 Villaviciosa de Odón, Spain; 10 Higher Institute of Medical Sciences of Havana Victoria de Girón, University of Medical Sciences of Havana, Havana 11600, Cuba

**Keywords:** spinal cord injury, gait, training

## Abstract

Introduction: Spinal cord injury is defined as the pathological process produced by any etiology affecting the spinal cord, which may alter motor, sensory, and/or autonomic function below the level of the lesion. The complexity of the neurological deficit and, therefore, the resulting clinical picture depends on the level of the lesion, the extent, and the affectation of the white or gray substance. This injury can totally or partially affect the ability to walk, and its highest priority with respect to mobility is to restore the ability to walk. All of which make the improvement of the methods used in their rehabilitation a top priority for health systems. Objective: The main objective of this study was to evaluate the effect of a gait training program for patients with spinal cord injuries. Material and Methods: A single-group, prospective cohort study was developed following the Strengthening the Reporting of Observational Studies in Epidemiology Guidelines (STROBE) at the International Center for Neurological Restoration of Siboney Playa (Havana, Cuba) from May 2020 to July 2021 with a sample of 30 patients by accidental or deliberate non-probabilistic sampling that met the expected inclusion criteria, who underwent a physical rehabilitation program for 8 weeks of work. Results: Statistically significant changes were observed in the overall course, by sex, by topographic level of lesion, and by functional class. Conclusions: The gait training program used produced significant changes in thoracic spinal cord injured patients regardless of the level of injury, sex, or functional class of the patient.

## 1. Introduction

Spinal cord injury (SCI) is a catastrophic event that represents a high health cost for health systems for the people and families who suffer from it [1] because this situation not only occurs in the acute phase of the injury but may or may not be maintained during their lifetime chronic diseases resulting from the injury [2]. According to a World Health Organization report, its incidence worldwide is between 40 and 80 cases per million inhabitants [3].

SCI is defined as the pathological process produced by any etiology that affects the spinal cord and may alter motor, sensory, and/or autonomic function below the level of the lesion [1,2]. The complexity of the neurological deficit and, therefore, of the resulting clinical picture depends on the level of the lesion, the extension, and the involvement of the white or gray matter [1,2,3].

Trauma is the leading cause of SCI, with 60% of cases occurring in developed countries and about 80% in developing countries; there, traumas are caused by stab wounds and firearms, traffic accidents, falls, diving into shallow water, sports, and work accidents [1,2].

Patients with spinal cord injury may lose all or part of their ability to walk, and their highest priority regarding mobility is to restore the ability to walk [4]. In addition, the loss of gait in patients is associated with the development of secondary disorders such as bone demineralization, muscle atrophy, or urinary tract infections [5].

Regarding gait in patients with spinal cord injury, according to studies [6,7,8], about 50% of patients with spinal cord injury walk, which may be determined by the fact that they use walking as their main mode of transport, that they use it only for therapeutic purposes or only to perform specific tasks in a standing position.

Despite the fact that different studies highlight the use of new technologies [9,10,11,12,13,14,15,16], it is necessary to develop work mechanisms that favor traditional training methods that are accessible to all and that meet the proposed objectives. Based on these foundations, the International Center for Neurological Restoration (CIREN) provides care to spinal cord injured patients with a multicomponent and intensive rehabilitation program that includes a methodology for gait training developed by the center’s specialists.

The main objective of this study was to demonstrate the efficacy of a gait training program for patients with spinal cord injuries.

## 2. Materials and Methods

### 2.1. Study Design

A single-group, prospective cohort study was developed following the Strengthening the Reporting of Observational Studies in Epidemiology Guidelines (STROBE) [17]. The study was conducted in accordance with the Declaration of Helsinki and approved by the Ethics Committee in Scientific Research, CEIO of CIREN 20 April 2020, by its Agreement No. 2 of Act No. 2. At all times, the confidentiality of the information was preserved, making responsible use of the data as established by current Cuban regulations.

### 2.2. Study Population

Between May 2020 and July 2021, 30 patients with spinal cord injuries admitted to the Clinic of Spinal Cord Injuries, Neuromuscular Diseases and Multiple Sclerosis of CIREN of Siboney Playa (Havana, Cuba) were selected by accidental or deliberate non-probabilistic sampling that met the inclusion criteria.

The inclusion criteria were:

- traumatic spinal cord injury older than 19 years;
- onset of more than 1 year;
- upper motor neuron injury with neurological level of injury from T1 to T12;
- and patients with the American Spinal Injury Association (ASIA) Impairment Scale (AIS) A, B, C, or D were included [5].

The exclusion criteria included:

- joint contracture of the lower extremity;
- fracture risk with severe osteoporosis;
- pressure injuries of the sacrum, ischium, or coccyx;
- cognitive impairment;
- and gait problems prior to spinal cord injury.

All selected patients who met the inclusion criteria underwent the rehabilitation program for spinal cord injured patients and a methodology for gait training of spinal cord injured patients described in the present study (Supplementary Materials).

### 2.3. Outcomes Measures

#### 2.3.1. Research Design According to the Gait Training Program Methodology

The following methodological steps were followed for the evaluation of the patients.

#### 2.3.2. Initial Evaluation of the Sample

All patients were evaluated at the Comprehensive Psychomotor Evaluation Laboratory (LEIS) of CIREN in Siboney Playa (Havana, Cuba) before the beginning of the treatment, where the corresponding scales “Waking Index for Spinal Cord Injury Version II” (WISCI II) and “Spinal Cord Independence Measure Version III” (SCIM) were applied. In the particular case of this scale, only the mobility item was taken. Once the scales were applied, the stratification process by functional classes was carried out (Table 1).

#### 2.3.3. Implementation of the Physical Rehabilitation Program

The selected sample was exposed to an intensive multifactorial rehabilitation program of 6 h daily, which included the methodology of walking for 8 weeks, in two daily sessions, with a frequency of 5 days a week with 1 hour daily and with the dosage of the load in correspondence with the functional group of each patient determined in the initial evaluation, with the indications and methodological requirements established for each stage of the rehabilitation process (Supplementary Materials S1: Physical Rehabilitation Program for patients with spinal cord injury).

#### 2.3.4. 1st Stage: General Preparation

At this stage, it was planned to improve muscle tone and joint mobility, try to reeducate the muscle groups affected by paralysis that have the potential for recovery, increase muscle strength in muscle groups above the injury, improve sitting balance, and achieve standing.

#### 2.3.5. 2nd Stage: Special Preparation

As conditions to pass to this stage of work, the patient must not have important articular limitations that allow them to achieve bipedestation. They must also be able to remain in the sitting position without losing balance and, in case of losing it, be able to recover it by themselves. The patient must present an improvement of at least 2 points on the SCIM III scale or have a total score greater than 20 points.

Muscle strength in the supralesional muscles must have increased by at least 2 kg in the case of triceps, biceps, or adductors; trunk flexion must be performed at an angle of less than 45°.

#### 2.3.6. 3rd Stage: Functional Preparation

To move to this stage of work, the patient must have been able to achieve standing in parallel without episodes of dysautonomia, be able to maintain at least one hour in the bipedal position, be able to perform transfers to and from the chair independently or with minimal assistance, move independently in the wheelchair, have an improvement of at least 2 points on the SCIM III scale or have a total score greater than 43 points.

Although activities from previous stages continue to be developed, the main objective is to achieve independent walking or walking with the aid of walking aids (walker, Canadian canes, cane) and with or without technical aids and to develop the patient’s endurance capacity.

### 2.4. Final Evaluation of the Sample

#### 2.4.1. Intervention Dosage

The study commenced with 30 patients who completed it without any limitations or negative health incidents that caused them to drop out. The adaptation of patient loads was guided by an initial evaluation using the WISCI II and SCIM III tools (Mobility item) to assess gait. Specific gait-related objectives were set for each patient, considering the rehabilitation period (all patients in the study underwent an 8-week program) and the patient’s upper limb and trunk muscle strength. Relative muscle strength was determined by the 10-repetition maximum in exercises such as elbow flexion, elbow extension, shoulder abduction, and horizontal shoulder adduction, as well as the maximal strength in the dorsal bar traction apparatus and bench press. The training involved working with loads ranging from 40% to 60% of the maximum strength, performing 5 sets per exercise, increasing repetitions (10, 12, and 15) before increasing weight, with a two-minute rest between sets.

Adaptation in skill development was planned for repetitions of 10, 15, and 20. Recovery time between exercise sets was 2 min, with daily sessions from Monday to Friday involving 4 skills per day. Load adjustments were made if a patient completed an activity or exercise with ease, increasing repetitions or weight to provide a suitable challenge.

To measure patient progress from a functional perspective, patients needed to improve by at least 2 points on the used tools (WISCI II and SCIM III), increase muscle strength, and execute gait skills with the appropriate technique and minimal effort to make it as natural as possible. In cases where patients encountered issues affecting load adaptation, adjustments were made (decreasing weight, repetitions, or activities) in line with the patient’s limitations, always in consultation with the medical team until the issue was resolved. 

#### 2.4.2. Study Evaluations

At the end of the last week of the established treatment period, the final evaluation was carried out. The same scales were used as in the initial evaluation, in the same place, and with the same external evaluators. The gait training was performed under the patients’ inpatient regime.

### 2.5. Statistical Analysis

For statistical analysis, the program R Ver. 4.1.3 (R Foundation for Statistical Computing, Institute for Statistics and Mathematics, Vienna, Austria) was used. The level of significance was established at *p* < 0.05. The Shapiro–Wilk test was used to test the distribution of the quantitative variables. The sample size was calculated with the pre-post-treatment from the WISCI, with the first 15 subjects recruited using a Student’s *t*-test for paired data. Quantitative variables were described with mean ± standard deviation and qualitative variables with absolute and relative values (%).

The presence of significant pre-post-treatment differences was explored using a linear mixed model with Restricted Maximum Likelihood (REML) in the quantitative outcome variables. Subjects were modeled as a random effect, and the time measurement was modeled as a fixed effect, including gender and injury level as covariates to evaluate their effect on treatment. The effect size was calculated for the pre-posttreatment effect using Cohen’s D, being defined as small (<0.5), moderate (0.5–0.8), and large (>0.8). In the case of the variable WISCI functional group, the presence of significant pre-post-treatment differences was analyzed using the Cochrane Q test and between both measurements based on gender or level of injury using the Cochrane–Mantel–Haenszel test. The effect size for the pre-post-treatment effect was calculated using Cochran’s eta square ($\eta_{Q}^{2}$) and was defined as small (<0.15), moderate (0.15–0.25), and large (>0.25).

## 3. Sample Size

Accepting a risk α < 0.05 and a power greater than 95% with 20% of estimated losses, a total of 30 patients are needed.

## 4. Results

### 4.1. Patient Flow and Principal Characteristics

A total of 30 patients participated with the eligibility criteria and were included in the physical rehabilitation program, balanced between 19 men (63%) and 11 women (37%) with an age of 35.33 ± 6.25 years and an average years of injury of 3 ± 1.13. Table 2 shows the clinical and demographic characteristics of the participants, with a maximum age difference between patients of 16 years and a maximum difference of 4 years for the time of lesion evolution.

### 4.2. Pre-Post-Treatment Results for All Outcome Variables

The presence of significant pre-post-treatment differences in all outcome variables is verified, with no differences between both measurements depending on the level of injury or gender, with higher post-treatment values in all cases and a greater final proportion of patients with a functional level I (43.3% vs. 0.0%) and II (56.7% vs. 40.0%) along with the absence of patients with level III (0.0% vs. 60.0%) (Table 3).

**Figure 1:**
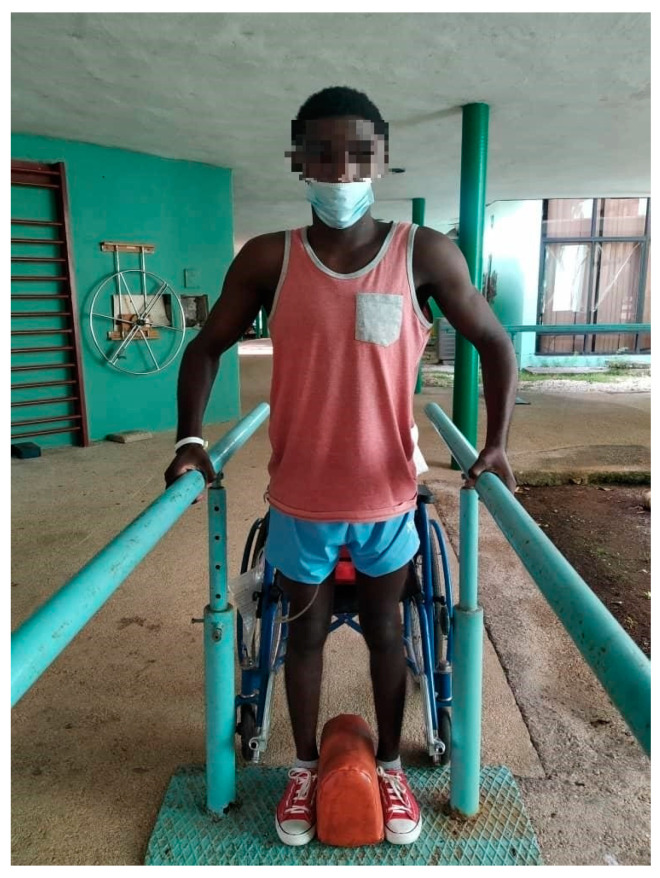
Spinal cord injured patient standing in parallel without orthosis. The objective of the activity was to improve posture. In this activity, the rehabilitator will observe the balance and weight distribution on both limbs and will use their hands to provoke imbalances in the patient so that they try to recover the proper position.

**Figure 2:**
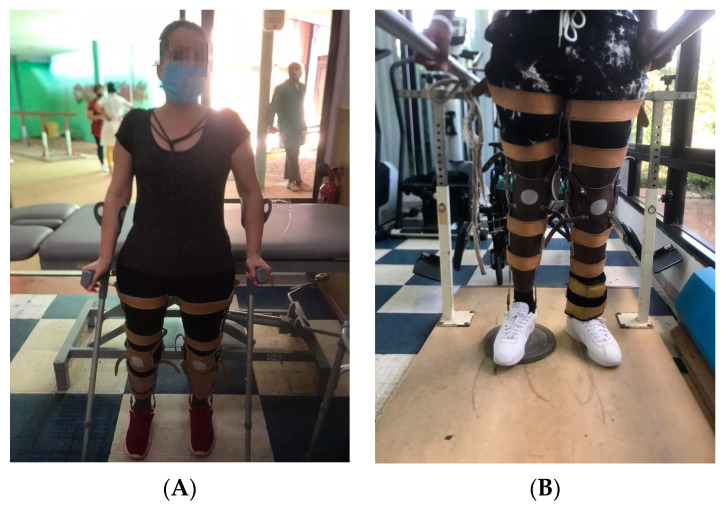
(**A**) Patient performing static balance with Canadian canes. The objective of the activity is to improve the posture with the Canadian canes. In this activity, the rehabilitator will observe the balance and the distribution of weight on both limbs and will use their hands to provoke imbalances in the patient so that they try to recover the proper position. In the case of the image, the patient was placed behind an exercise table to give her confidence so that in case of imbalance, she could use her buttocks to support herself on the stretcher and recover the initial posture. (**B**) Spinal cord injured patient standing in parallel with orthosis with the opposite limb to the one performing the movement resting on a 3 cm high surface. The objective of the activity is to train the limb that is in suspension. In this activity, the patient should perform the activities indicated by the rehabilitator (displacement of the foot to the front and back, elevation of the pelvis, one side and the other, and unloading of weight). The proper posture should be observed during the activity.

It was observed that in the final evaluation, all patients managed to walk at least in a walker, that 28 of the 30 patients changed functional groups, and only 2 of them remained in the same group (patients 27 and 30). However, this did not mean that there were no functional changes in terms of their group and proof of this is that both entered the rehabilitation service with a gait with orthosis and walker with supervision up to 10 m and at the end of the physical rehabilitation program they were able to move more than 100 m with Canadian canes and without supervision. The change was reflected in the distance traveled, safety, and assistance moving from the walker to the Canadian canes. In addition, patient 30 was able to walk up and down three steps (Figure 1 and Figure 2).

All figures and videos (Supplementary Materials S2) correspond to activities that were used in stage 3 of the program.

**Figure 3:**
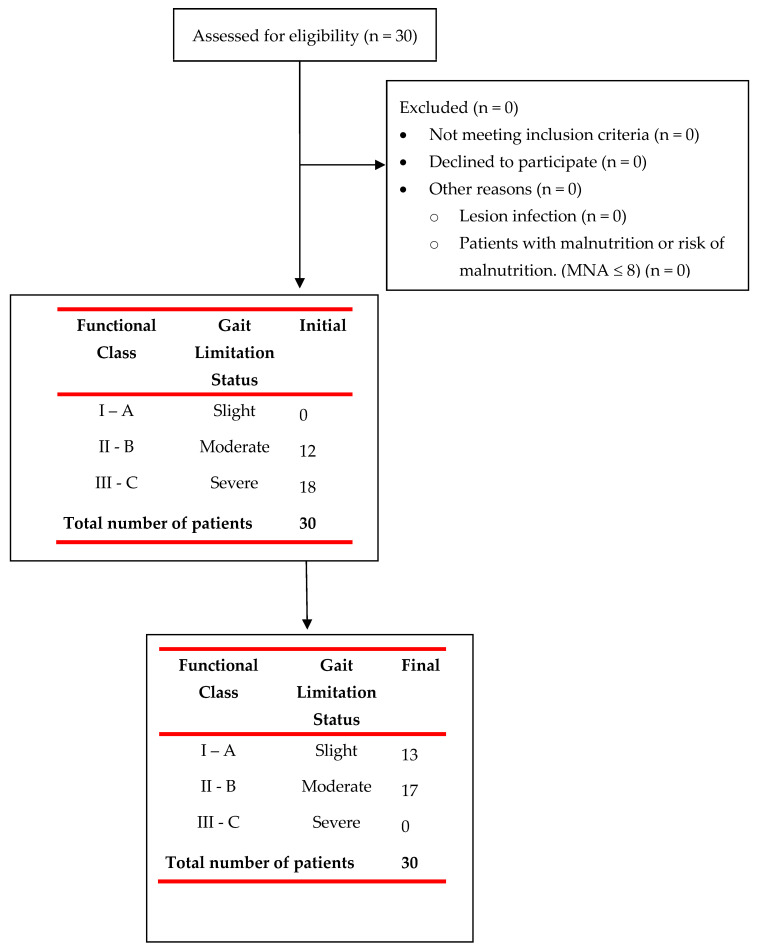
Flowchart of patient recruitment for the study and their initial and final evaluation.

Figure 3 shows the Gait Limiting Status in which the participants were prior to enrolling in this study and the grades in which they finished.

## 5. Discussion

The gait training program used produced significant changes in thoracic spinal cord injured patients regardless of the level of injury and sex. The program itself included passive and active joint mobilization, strength, gait and balance training, and re-education of daily activities, and aimed to improve physical health and gait independence. 

All variables included improved with small to large effect sizes. Notably, the largest effect sizes were found for WISCI scores and indoor/outdoor mobility. In addition, WISCI improved from 4.3 (±3.02) to 10.43 (±1.74), which relates to real differences in the clinical setting based on previously published studies [14,18,19,20,21,22]. 

Gait training is essential in patients after SCI, and different forms of gait training have been proposed for years. A very recent published network meta-analysis revealed that functional electrical stimulation tended to be the most effective gait training method for walking speed and distance, followed by treadmill, robot-assisted gait training, and conventional physical therapy [14].

Our results were in concordance with others who used robotic systems for gait reeducation in patients with incomplete spinal cord injury, reporting increases in the WISCI index after gait training [19], as well as with others who found improvements in mobility after rehabilitation in patients with spinal cord injury [20,21,22]. 

Taking into account the number of scientific articles that relate gait training to the use of new rehabilitation methods, it can be inferred that the attention given to conventional gait training methods is scarce, but not the same for those articles that focus on the use of new technologies [23,24,25,26,27].

However, the results obtained by the introduction of new technologies alone are not sufficient to achieve adequate gait training, with the combination of conventional therapy combined with new technologies contributing to a significant improvement in gait parameters [26].

In fact, as detailed in a recent systematic review, robot-assisted gait training alone fails to improve such important parameters in gait rehabilitation as walking distance, walking speed, and leg strength [27]. To achieve an improvement in these indicators, it is necessary to improve the gait patterns so that as the gait becomes more efficient in terms of its performance, the patient will have less energy expenditure, which will allow him to increase the distance and speed to be covered. In addition, as shown in the present study, during the last phase of the gait rehabilitation program, auxiliary means such as lower limb weights are used in order to make the gait work more complex and to strengthen the muscles used to perform the movement.

Among the strengths of this study is the direct work of the rehabilitator with the patient with the corresponding control and evaluation of the therapy that is adapted according to the real conditions of each patient, although the walking speed was not measured. However, a recent systemic review in patients with spinal cord injury has found that WISCI is more reflective of rehabilitation characteristics when robotic gait training is performed, so they recommend performing studies with different locomotor training approaches and targets that assess function and performance rather than speed [28].

It should be clarified that during the gait training program of the present study, the principles of sports training adapted to the therapy were used, in which the development of physical abilities plays an important role in the establishment of static and dynamic gait patterns. The physical capacities that were developed during the gait training program used were strength and endurance, which have been addressed with satisfactory results in other studies that have been developed in the rehabilitation of spinal cord-injured patients [29,30].

Although no cardiovascular variables were collected in the present study, the only fact of staying for 8 weeks in the gait training program with high demand (decreasing sitting time, reaching distances of 100 m and more, with an effective working time of more than 40 min, and with a high work intensity) is thought to have increased the constant blood flow effort, in agreement with what was observed by Faulkner et al., who evaluated the effects of assisted gait training on central vascular health in 12 patients with spinal cord injury [31].

Among the aspects that coincide or not with other studies, we can find the similarity of the gait training time for 8 weeks by several authors [19,32,33,34,35], which seems to indicate that it is approximately the time necessary to develop the gait ability.

A study using a robotic system for gait reeducation in patients with incomplete spinal cord injury reported an increase in the WISCI index from 5.5 to 12.5 (*p* < 0.001) with a mean of 7 points, [36] while in the present study, it was 4.30 and 10.43 (*p* < 0.001) with a mean of 6.13 points. These results are also very similar to those reported by Gupta et al. [37], who found a mean number of points for the WISCI of 6.2 points. Regarding the mobility item of the SCIM III scale, the physical rehabilitation program used in the present study was superior to that observed in a multicenter study by Baunsgaard et al. [34]. Likewise, the results of the present study for the same variables are superior to those achieved by a study that evaluated the influence of dynamic parapodial training on the improvement of the patients’ gait motor functions and which reported changes in the control and experimental groups of 0.36 and 2.29 points in the WISCI, respectively [38].

Overall, the data obtained tend to favor motor function and mobility, which corresponds to a study by Mıdık et al. [39]. 

### Limitations and Future Directions

The results achieved in the study show a functional improvement in all patients when they are able to walk, but there are some limitations present in the study. First, the sample used was not conditioned to a certain level of neurological impairment. Second, certain components that interfere with walking, such as spasticity, were not analyzed, as other studies did [25,40]. Third, gait speed and distance between steps were not controlled in the present study as other studies did [41,42]. However, it was not an objective to be evaluated in this study. Fourth, we acknowledge that we did not include a control group, and sample recruitment was not randomized, which could introduce some bias. Therefore, the need for similar studies in a larger group of patients is suggested. Based on these limitations, the generalizability of the results is limited.

## 6. Conclusions

The gait training program used produced significant changes in thoracic spinal cord injured patients regardless of the level of injury, sex, or functional class of the patient. Its application in rehabilitation centers that attend to this type of patient may be relevant provided that they have the necessary means and their professionals are properly trained for this purpose.

## Figures and Tables

**Table 1:** Functional group based on gait, CIREN, 2021.

Functional Class	WISCI-II	SCIM III (Mobility)	Gait Limitation Status
I—A	10 & >	29 & >	Slight
II—B	6–9	18–28	Moderate
III—C	0–5	0–17	Severe

WISCI-II—Waking Index for Spinal Cord Injury Version II. SCIM III—Spinal Cord Indepence Measure Version II (mobility item). Gait limitation status according to the author. Methodological guidelines according to the procedure.

**Table 2:** Clinical and demographic characteristics of the participants.

*n*		30
Age		35.33 ± 6.25
Gender, *n* (%)	Female	11 (36.7)
	Male	19 (63.3)
Time of evolution (years)		3.03 ± 1.13
Topographic level, *n* (%)	T1	2 (6.7)
	T10	6 (20.0)
	T11	1 (3.3)
	T12	4 (13.3)
	T4	3 (10.0)
	T5	2 (6.7)
	T6	5 (16.7)
	T7	2 (6.7)
	T8	5 (16.7)
Injury level, *n* (%)	High (T1–T6)	12 (40.0)
	Low (T7–T12)	18 (60.0)
ASIA, *n* (%)	A	16 (53.3)
	B	14 (46.7)

Data expressed as mean ± standard deviation or with absolute and relative values (%). ASIA—American Spinal Injury Association.

**Table 3:** Outcome variables.

		Post-Treatment	Pre-Treatment	Average Difference (95%CI)	Measurement Time ^a^	Measurement Time: Injury Level ^a^	Measurement Time: Gender ^a^	Effect Size (95%CI)
WISCI		10.43 ± 1.74	4.30 ± 3.02	6.133 (4.854, 7.413)	F(1, 27) = 145.805, *p* < 0.001	F(1, 27) = 0.527, *p* = 0.474	F(1, 27) = 1.087, *p* = 0.306	2.102 (0.823, 5.839) *
WISCI functional group, *n* (%)	I	13 (43.3)	0 (0.0)		X2(1) = 28, *p* < 0.001	M2(2) = 0.044, *p* = 0.978	M2(2) = 0.239, *p* = 0.887	0.933 ↑
	II	17 (56.7)	12 (40.0)					
	III	0 (0.0)	18 (60.0)					
Mobility in bed		5.93 ± 0.37	4.40 ± 1.22	1.533 (1.061, 2.006)	F(1, 27) = 44.992, *p* < 0.001	F(1, 27) = 2.034, *p* = 0.165	F(1, 27) = 0.028, *p* = 0.868	1.048 (0.394, 3.61) *
Bed chair transfer		2.00 ± 0.00	1.87 ± 0.35	0.133 (0.004, 0.262)	F(1, 53.989) = 2.605, *p* = 0.112	F(1, 53.989) = 2.969, *p* = 0.091	F(1, 53.989) = 0.14, *p* = 0.71	0.062 *
Transfer chair toilet bath		2.00 ± 0.00	1.77 ± 0.43	0.233 (0.073, 0.394)	F(1, 54) = 7.449, *p* = 0.009	F(1, 54) = 0.505, *p* = 0.48	F(1, 54) = 0.186, *p* = 0.668	0.354 (0, 1.238) *
Indoor mobility		4.43 ± 0.50	2.80 ± 1.00	1.633 (1.222, 2.045)	F(1, 27) = 112.461, *p* < 0.001	F(1, 27) = 0.06, *p* = 0.808	F(1, 27) = 0.195, *p* = 0.662	2.019 (0.759, 4.01) *
Mobility over moderate distances		4.43 ± 0.50	2.73 ± 0.98	1.7 (1.294, 2.106)	F(1, 27) = 206.136, *p* < 0.001	F(1, 27) = 0.614, *p* = 0.44	F(1, 27) = 1.013, *p* = 0.323	2.562 (0.854, 5.461) *
Outdoor mobility		4.23 ± 0.43	2.27 ± 0.69	1.967 (1.668, 2.266)	F(1, 27) = 222.924, *p* < 0.001	F(1, 27) = 3.691, *p* = 0.065	F(1, 27) = 1.118, *p* = 0.3	3.203 (1.575, 6.817) *
Handling on stairs		0.97 ± 0.93	0.07 ± 0.25	0.9 (0.543, 1.257)	F(1, 27) = 28.535, *p* < 0.001	F(1, 27) = 0.206, *p* = 0.653	F(1, 27) = 0.175, *p* = 0.679	1.26 (0.392, 2.843) *
Chair car transfer		2.00 ± 0.00	1.30 ± 0.60	0.7 (0.477, 0.923)	F(1, 32.33) = 38.912, *p* < 0.001	F(1, 32.33) = 0.09, *p* = 0.766	F(1, 32.33) = 0.608, *p* = 0.441	1.356 (0.193, 3.645) *
Floor chair transfer		0.73 ± 0.91	0.03 ± 0.18	0.7 (0.356, 1.044)	F(1, 27) = 15.305, *p* = 0.001	F(1, 27) = 0.02, *p* = 0.89	F(1, 27) = 0.078, *p* = 0.782	0.712 (0.024, 1.798) *
Subtotal		26.73 ± 3.14	17.23 ± 3.93	9.5 (7.66, 11.34)	F(1, 27) = 397.559, *p* < 0.001	F(1, 27) = 0.319, *p* = 0.577	F(1, 27) = 0.242, *p* = 0.627	3.843 (1.717, 11.425) *

Data expressed as mean ± standard deviation or with absolute and relative values (%). WISCI—Walking Index Spinal Cord Injury. 95%CI—95% confidence interval. *: Group Cohen’s D effect size. ↑: $\eta_{Q}^{2}$ effect size. ^a^ significant if *p* < 0.05 (shown in red).

## Data Availability

The data presented in this study are available on request from the corresponding authors. The data are not publicly available due to ethical restrictions.

## Supplementary Materials

The following supporting information can be downloaded at https://www.mdpi.com/article/10.3390/jcm12237208/s1. Supplementary Materials S1: physical rehabilitation program for patients with spinal cord injury. Supplementary Materials S2: description of videos of the Physical Rehabilitation Program for patients with spinal cord injury S1–S6. Video S1: spinal cord injured patient training the parallel gait with a cane. This activity is performed with the objective of helping the patient adapt to the use of Canadian canes. Its realization in parallel gait provides greater safety to the patient, allowing them to perform in a safe environment that, in case of any imbalance, can be helped by the parallel gait, besides focusing on the mastery of one hemibody (the one supporting the cane) because the other one has been mastered in previous stages. Video S2: spinal cord injured patient training the parallel gait with the use of weights on the lower limbs. This activity should be performed once the patient has mastered the ability to walk without technical errors; overweights are added to the lower limbs in order to force the patient to a greater elevation of the hemipelvis during walking and contribute to the strengthening of the musculature that contributes to the activity. Video S3: spinal cord injured patient walking with a walker outdoors. It was developed with the aim of perfecting the skills in handling the walker outdoors (wide places in which displacement activities are included: on uneven surfaces, ascents, and descents). In addition to functional walking skills, the patient’s endurance capacity must be developed here. Video S4: patient walking with a walker in the gymnasium. It was developed with the aim of perfecting the skills in the handling of the walker in confined spaces. The objective is to develop skills in the handling of the walker in more closed environments, in which the patient has to deal with different barriers that make it difficult to move. The aim is to improve the patient’s ability to respond to the contingencies that arise. Video S5: spinal cord injured patient training on the thera trainer. The objective is to improve the infralesional musculature and thus contribute to achieving walking without orthosis or decreasing its level. Video S6: spinal cord injured patient receiving electrical stimulation. The objective is to improve the infralesional musculature in the affected muscles and contribute to the gait training process in its different variants. This procedure is performed by a specialist in physical medicine and rehabilitation. Ref. [43] is cited in the Supplementary Materials S1.
