# Supplementary material for: Effects of a Gait Training Program on Spinal Cord Injury Patients: A Single-Group Prospective Cohort Study
Source: J Clin Med. 2023 Nov 21;12(23):7208. doi: 10.3390/jcm12237208 (PMC10707500; doi:10.3390/jcm12237208)
Supplement: Supplementary file 1 [file jcm-12-07208-s001.zip › S_M_1_Physical rehabilitation program.pdf]

**Rehabilitation program for spinal cord injured patients**

The rehabilitation program is aimed at spinal cord injured patients who are in the second stage of the Neurological Restoration Program at the International Center for Neurological Restoration and is planned for 6 hours of work Monday through Friday and 3.5 hours on Saturdays.

It is important to note that all patients undergo a week of medical evaluation by the multidisciplinary team before starting rehabilitation to establish or confirm the diagnosis and analyze the starting point to establish the guidelines to be followed by each specialty.

With this in mind, a work program was created with general and specific objectives that are personalized according to the patient's current condition, functional characteristics and type of injury. Taking into account the above elements, a personalized rehabilitation program was designed for the patients in the research.

The work program was conceived in three stages of work, the methodology for gait training is introduced from the second stage.

The SCIM scale version III is used to ensure the control, evaluation and follow-up of the rehabilitation program.[1]

**GENERAL OBJECTIVES OF THE EMPLOYEE PROGRAM**

- Modulation of muscle tone.
- Increase basic physical capacities (strength, endurance, speed, flexibility).
- Increase physical coordination skills (balance and coordination).
- Obtain the maximum functional independence (ADL) possible.

**SPECIFIC OBJECTIVES OF THE EMPLOYEE PROGRAM**

- Neuromuscular reeducation of muscle groups affected by paralysis that present potential for recovery.
- Increase muscle strength in muscle groups above the injury.
- To develop endurance capacity.

---

[1] Zarco-Perinan MJ, Barrera-Chacon MJ, Garcia-Obrero I, Mendez-Ferrer JB, Alarcon LE, Echevarria-Ruiz de Vargas C. Development of the Spanish version of the Spinal Cord Independence Measure version III: cross-cultural adaptation and reliability and validity study. Disability and rehabilitation. 2014;36(19):1644-51.

- Improving sitting and standing balance
- Make transfers .
- Achieving independent walking or with the aid of walking aids (walker, Canadian canes, cane) and with or without technical aids.

**Work program used**

**1st stage General preparation.**

At this stage it was planned to improve muscle tone, the joint mobility, try to reeducate the muscle groups affected by paralysis that have potential for recovery, increase muscle strength in muscle groups above the injury, improve sitting balance and achieve standing.

In order to meet the above objectives, certain activities were developed, such as:

- Passive, assisted and resisted mobilizations of the upper and lower limbs.

Passive manipulations of the lower limbs were performed in all patients for 15 minutes per session, and in the case of patients evaluated as ASIA B, C and D, assisted mobilizations were used in the last third to complete the range of motion.

Resisted mobilizations were used in lower limbs in those patients who had grade 4 muscle strength.

To help modulate muscle tone, the following measures were adopted for seated work.

- ✓ For patients with lower limb spasticity in flexion, place the patient with knees extended.
- ✓ For patients with lower limb spasticity in extension, place the patient in a seated position with knees bent in front.
- ✓ For patients with lower limb spasticity in abduction, place the patient in a seated position with knees bent and straddled.

The free exercises for the general physical development planned were established based on the segment to be developed and included all the possible movements of each segment treated.

**Free exercises**

- Head
- Upper limbs

- Trunk
- Lower limbs.

Free exercises were mainly used in warm-up and limbs with grade 2 muscle strength.

These exercises were also used in the work of muscle development, but with the use of dumbbells and overweights.

In order to establish the dosage of the muscle development exercises, it was established based on the estimation of the relative maximum strength of 10 repetitions maximum.

For each planned exercise, 10 series of 10 repetitions with a weight (approximately 65% of the estimated maximum strength) with a rest of 2 minutes between each series are proposed. Later in the second week, depending on the adaptation to the workload, it will be increased to 12 repetitions maintaining the rest time and the series, then it will be increased to 15 repetitions, also maintaining the rest time and the series. After this, the cycle will begin again increasing the work weight with 10 series of 10 repetitions.

As special exercises to develop complementary skills in gait training were performed:

- On the mattress in the supine position, turn alternately to both sides.
- On the mattress in supine decubitus hip extension, with roller.
- On the mattress in the supine position turn to prone position
- On the stretcher in supine decubitus w/ leg flexed, lateral movements of the same w/ overweight.
- On the supine table with both legs flexed, perform hip extensions with overweight.
- On the supine couch with both legs flexed on a roller, perform hip extensions w/o overweight.

For these exercises, the same methodology described above will be used. The equipment for the development of muscular strength is presented below.

- Horizontal Press
- Multi-strength (flexion and extension will be performed to strengthen pectoral, shoulder and paravertebral muscles).
- High pulley (halon will be performed at the front and at the back)
- Quadriceps Bench (Knee extension)
- Biceps femoris bench (Knee flexion)

- Multifunctional muscle trainer For training the thigh and gluteus muscles, especially the anterior thigh, gluteus maximus and calf muscles.
- Back stabilizer / arm trainer (dual function) For training back extensor muscles, shoulder, arm flexors, pectorals and arm extensors.
- Leg flexion/extension equipment (dual function) Knee flexion and extension.
- Trunk flexion / extension. Trunk extension / flexion equipment (straight and upper lateral abdominals, dorsals).
- Hip flexion/extension equipment (dual function) For hip flexion/extension and foot adductor/abductor training.
- Explosive pulley system. For universal use and training of the shoulder girdle, upper and lower extremities.
- Balance Trainer. For balance and equilibrium training.

A working time of 60 minutes per day was used to develop activities such as free exercises for coordination and balance, ending with ball throwing activities in different directions and speeds to increase the complexity of the activity.

- Dual-function bike. THERA-vital bike and upper limb trainer.

It was used only for lower limb activities at a dosage of 15 minutes per day in patients evaluated for ASIA B, C and D.

**<u>2 $^{nd}$ Stage Special preparation.</u>**

As conditions to pass to this stage of work, the patient must not have important articular limitations that allow him/her to achieve bipedestation, he/she must also be able to remain in the sitting position without losing balance and in case of losing it, be able to recover it by him/herself. The patient must present an improvement of at least 2 points on the eSCIM III scale or have a total score greater than 20 points.

Muscle strength in the supralesional muscles must have increased by at least 2 kg in the case of triceps, biceps or adductors, trunk flexion must be performed at an angle of less than 45°.

The objectives to be developed in this stage will be aimed at:

- Modulate muscle tone
- Improve joint mobility

- Neuromuscular reeducation of muscle groups affected by paralysis that present potential for recovery.
- Increase muscle strength in the muscle groups above the lesion
- To develop endurance capacity.
- Improve standing balance
- Performing transfers (all to and from the chair)

**Activities**

The activities of the previous stage are continued to modulate muscle tone, joint mobility, neuromuscular reeducation and muscle strength.

Resistance work is introduced, standing balance work is incorporated and the complexity of the coordination exercises is increased, and the work on gait patterns **is** started in 4 points

Displacement in the wheelchair on the track

**Transfers**

Transfer training will be performed from and to the chair to ensure compliance with all activities of daily living. It is important to note that in order to perform this work the patient must have achieved adequate trunk strength to maintain sitting balance and muscle strength in the upper limbs with emphasis on the triceps.

As a previous activity, the elbow extension should be worked from the chair to detach the support of the buttocks.

The 4-point exercises are prefunctional gait patterns that aim to replicate the bipedal gait patterns from that position and are the fundamental basis of gait work because from these elements the fundamental bases of gait are created and the technical errors that cannot be solved at this stage will be carried over to gait training in the bipedal position.

The training of the 4 points used will be 60 minutes per day, which makes it the main activity of the stage. At least one week of work is recommended, but the recommended time is 2 weeks.

**Activities in 4 points**

- That the patient adopts and maintains the 4-point support position.
- From the 4-point position, try to unbalance the patient.
- From the 4-point position, the patient performs hip movements to the front and back (with or without overweight).

Note: Overweights will be tied around the hip.

- From the 4-point position, the patient performs lateral hip movements (with or without overweight).

Note: Overweights will be tied around the hip.

- From the 4-point position, the patient performs elevation of the hemipelvis in place (with or without overweight).

Note: Overweights will be tied around the thigh.

- From the 4-point position, the patient performs displacement to the sides of the hemipelvis in place (with or without overweight).

Note: Overweights will be tied around the thigh.

- From the 4-point position, the patient performs a step forward and backward (with or without overweight).

Note: Overweights will be tied around the thigh.

- Crawling (with or without overweight).

Note: Overweights will be tied around the thigh.

## 3 rd Stage. Functional preparation.

To move to this stage of work the patient must have been able to achieve standing in parallel without episodes of dysautonomia, must be able to maintain at least one hour in the bipedal position, must be able to perform transfers to and from the chair independently or with minimal assistance, move independently in the wheelchair, must have an improvement of at least 2 points on the SCIM III scale or have a total score greater than 43 points.

Although activities from previous stages continue to be developed, the main objective is to achieve independent walking or with the aid of walking aids (walker, Canadian canes, cane) and with or without technical aids and to develop the patient's endurance capacity.

**Activities**

The activities of the previous stage are continued to modulate muscle tone, joint mobility, neuromuscular reeducation and muscle strength.

Walking work is introduced with attachments and resistance work is deepened.

- Static and dynamic gait patterns in parallel gait.
  - ✓ Hip flexion and extension
  - ✓ Weight downloads
  - ✓ Step forward and backward
  - ✓ Passage to the sides
  - ✓ Parallel march to the front
  - ✓ Lateral march
- Training with the walker or Canadian canes).
  - ✓ Maintaining the standing position
  - ✓ Perform hip movements in all directions.
  - ✓ Step forward and backward
- Walking with gait aids (with or without long or short orthoses, equine corrective braces, stabilizing knee braces).
- Going up and down stairs.
- Marching with alternating leg lifts in place.
- Exercise bike
- Climber
- Walking without poles.
- March through irregular terrain
